# Räumlich-zeitliche Psychopathologie – deutsche Version der Scale for Space and Time Experience in Psychosis (STEP): Ein validiertes Messinstrument zur Erfassung des räumlichen und zeitlichen Erlebens bei psychotischen Störungen

**DOI:** 10.1007/s00115-023-01519-y

**Published:** 2023-07-10

**Authors:** Dusan Hirjak, Jonas Daub, Geva A. Brandt, Maria Krayem, Katharina M. Kubera, Georg Northoff

**Affiliations:** 1 Klinik für Psychiatrie und Psychotherapie, Zentralinstitut für Seelische Gesundheit, Medizinische Fakultät Mannheim, Universität Heidelberg, J5, 68159 Mannheim, Deutschland; 2 Zentrum für Psychosoziale Medizin, Klinik für Allgemeine Psychiatrie, Universität Heidelberg, Heidelberg, Deutschland; 3 Mind, Brain Imaging and Neuroethics Research Unit, The Royal’s Institute of Mental Health Research, University of Ottawa, Ottawa, ON Kanada

**Keywords:** Psychotische Störungen, Schizophrenie, Affektive Störungen, Phänomenologie, Differenzialdiagnostik, Psychotic disorders, Schizophrenia, Affective disorders, Phenomenology, Differential diagnostic

## Abstract

Historische Autoren (u. a. Ludwig Binswanger und Eugène Minkowski) postulierten, dass das Erleben von Patient*innen mit psychotischen Störungen durch „Zeitfragmentierung“ charakterisiert ist. Aus klinischer Praxis ist bekannt, dass Patient*innen mit psychotischen Störungen auch unter Schwierigkeiten der räumlichen Wahrnehmung (z. B. Anomalien im Erleben zwischenmenschlicher Distanz und räumlicher Orientierung) leiden. Obwohl diese Veränderungen zu einer Art Abkopplung von der Realität, zu erheblichem Leidensdruck der Betroffenen und Schwierigkeiten im therapeutischen Prozess führen können, wurde das abnorme Erleben von Raum und Zeit bei psychotischen Störungen bisher nicht ausreichend untersucht. Eine mögliche Ursache ist das Fehlen geeigneter und standardisierter Instrumente, die das Erleben von Raum und Zeit bei Patient*innen mit psychotischen Störungen quantifizieren. Auf Basis eines innovativen Konzepts, der sog. „Räumlich-zeitlichen Psychopathologie“ (*„*spatiotemporal psychopathology“, STPP), wurde erstmalig eine Skala zur systematisch-quantitativen Erfassung des räumlichen und zeitlichen Erlebens bei Patient*innen mit psychotischen Störungen entwickelt. In dieser Arbeit wird nun die deutsche Version der Scale for Space and Time Experience in Psychosis (STEP) vorgestellt. Die englische Originalversion der STEP erfasst in 25 Items verschiedene räumliche (14 items) und zeitliche (11 items) Erlebensphänomene. Die STEP zeigt sowohl eine hohe interne Konsistenz (Cronbach’s *α* = 0,94) als auch einen signifikanten Zusammenhang mit der Positive and Negative Syndrome Scale (PANSS; *p* < 0,001). Zusammenfassend stellt die hier vorgelegte deutsche Version der STEP-Skala ein wichtiges Messinstrument im deutschsprachigen Raum zur Erfassung des räumlichen und zeitlichen Erlebens bei psychotischen Störungen dar.

**Zusatzmaterial online:**

Zusätzliche Informationen sind in der Onlineversion dieses Artikels (10.1007/s00115-023-01519-y) enthalten.

## Abstract (en)

Historical authors (e.g., Ludwig Binswanger and Eugène Minkowski) postulated that the experience of patients with schizophrenia is characterized by time fragmentation. From a clinical perspective, patients with schizophrenia also suffer from difficulties in spatial perception (e.g., abnormalities in the experience of interpersonal distance and spatial orientation). Although these changes can lead to a serious detachment from reality, to considerable suffering of the affected persons and to difficulties in the therapeutic process, the abnormal experience of space and time in psychotic disorders has not yet been sufficiently investigated. One possible reason is the lack of appropriate and standardized instruments that quantify the experience of space and time in patients with psychotic disorders. Based on an innovative concept, the so-called spatiotemporal psychopathology (STPP), a clinical rating scale for the systematic-quantitative assessment of spatial and temporal experience in patients with psychotic disorders was developed. This article presents the German version of the Scale for Space and Time Experience in Psychosis (STEP). The original English version of the STEP measures different spatial (14 phenomena) and temporal (11 phenomena) phenomena in 25 items. The STEP shows both a high internal consistency (Cronbach’s alpha = 0.94) and a significant correlation with the Positive and Negative Syndrome Scale (PANSS; *p* < 0.001). In summary, the German version of the STEP scale presented here represents an important instrument in the German-speaking countries for the assessment of spatial and temporal experience in patients with psychotic disorders.

## Hintergrund

Die Psychopathologie ist der empirische und theoretische Rahmen, in dem das subjektive Erleben, die klinischen Symptome und das beobachtbare Verhalten psychiatrischer Patient*innen beschrieben, kategorisiert und klassifiziert werden können [1–3]. In den letzten Jahrzehnten haben sich in der wissenschaftlichen Psychiatrie drei Ansätze zur Erforschung psychopathologischer Symptome bei Patient*innen mit psychischen Erkrankungen etabliert: (1) Das funktionsbasierte Modell der psychopathologischen Symptome nimmt an, dass psychopathologische Symptome auf abnormen Strukturen und Funktionen der entsprechenden Gehirnregionen/-netzwerke beruhen [4–9]. (2) Das kognitionsbasierte Modell der psychopathologischen Symptome postuliert, dass das subjektive Erleben auf kognitiven Funktionen höherer Ordnung und deren neuronalen Mechanismen beruht und daraus abgeleitet wird [3, 10, 11]. (3) Dem phänomenologischen Modell zufolge ist das subjektive Erleben von Patient*innen mit Depression oder Schizophrenie sowie das Erleben ihres basalen Selbst, ihres Körpers/Leibes und ihrer Welt verändert [12, 13]. Die phänomenologische Psychopathologie erfasst als analysierende sowie deskriptive Grundlagenwissenschaft der Psychiatrie die krankhaften Erscheinungsweisen der menschlichen Seele und hat Anfang des 20. Jahrhunderts mit der *Allgemeinen Psychopathologie* [14] von Karl Jaspers (1883–1969) Eingang in die klinische Praxis gefunden. Auf dem Gebiet der Philosophie wurde Jaspers unter anderem von Edmund Husserl (1859–1938) und seiner phänomenologischen Theorie geprägt. Nach Jaspers leistet die deskriptive Phänomenologie eine wichtige und grundlegende Basis für die Psychopathologie. Sie ist dadurch charakterisiert, dass sie sich um eine anschauliche Vergegenwärtigung der subjektiven Erlebnisse von Patient*innen mit psychischen Erkrankungen im Vollzug eines empathischen Verstehens ihres seelischen Leidens und eines Sichhineinversetzens in deren pathologisches Erleben und Empfinden bemüht [15]. Dabei fällt dem subjektiven Erleben eine prominente Rolle zu, indem es als eine basale Störung psychischer Erkrankungen aufgefasst wird [3, 16, 17]. Die phänomenologische Methode erfasst daher, gegenüber der kriteriologischen Diagnostik der modernen Klassifikationssysteme, die veränderten subjektiven Erlebens- und sozialen Verhaltensweisen im Gesamtkontext der Patient*innen. Daher ist die phänomenologische Diagnostik mehr Person-, die symptomatologische Diagnostik mehr *krankheitsorientiert* [18, 19].

Obwohl die o. g. drei Modelle wesentlich zum Verständnis der Neurobiologie, Neuropsychologie und Phänomenologie psychopathologischer Symptome beigetragen haben, ist es ihnen bisher nicht gelungen, die erzielten Ergebnisse in die neuen Klassifikationssysteme psychischer Störungen DSM‑5, ICD-10 und ICD-11 zu integrieren. Die Klassifikation psychischer Erkrankungen nach DSM‑5, ICD-10 und ICD-11 erfolgt nach fest vorgegebenen Kriterien, reduziert seelisches Leiden auf einfache, historisch-theoretische psychopathologische Konzepte und hat somit zum Ziel, die Reliabilität der psychiatrischen Diagnosen zu steigern. Es entsteht der Eindruck, dass die psychischen Erkrankungen exakt abgrenzbare und präzise definierbare seelische Störungen im Sinne somatischer Krankheiten sind. Der Schwerpunkt der manualisierten psychiatrischen Diagnostik basiert nicht auf den subjektiven Erlebnissen oder neurobiologischen Parametern von Patient*innen, sondern vordergründig auf relativ einfach zu beobachtenden und explorierenden Merkmalen ihres Verhaltens [20]. Darüber hinaus spielen derzeit weder strukturelle oder funktionelle Bildgebungsparameter noch neuropsychologische Aspekte für die Klassifizierung, Diagnose oder Therapie von psychischen Erkrankungen eine zentrale Rolle. Möglicherweise kann die Kombination klinischer und bildgebender Daten in der Zukunft zur Vorhersage des Krankheitsverlaufs beitragen. Die rezente Studie von Koutsouleris et al. [21] weist darauf hin, dass der Übergang in eine Psychose in einem breiteren Risikospektrum vorhergesagt werden kann, indem die Risikoeinschätzungen von auf struktureller Bildgebung basierten Algorithmen und Klinikern sequenziell integriert werden.

Wir argumentieren, dass ein alternativer Ansatz, der auf den Konzepten des räumlichen und zeitlichen Erlebens historischer Autoren (z. B. Ludwig Binswanger (1881–1966) und Eugène Minkowski (1885–1972)) und der modernen computergestützten Bildgebung beruht und das subjektive Erleben, die psychopathologischen Symptome und die Gehirnregionen/-netzwerke der Patient*innen einbezieht, dazu beitragen wird, die Limitationen der o. g. drei Modelle zu überwinden und Antworten auf die beiden Kernfragen der Psychiatrie zu geben. In der jüngsten Vergangenheit wurde zunehmend der Wunsch artikuliert, das subjektiv veränderte Raum- und Zeiterleben mit objektiv messbaren Parametern zu verbinden und gemeinsam zu untersuchen [22–25].

In englischer Sprache gibt es bereits mehrere validierte Instrumente, welche das veränderte Raum- und Zeiterleben von Patient*innen mit Schizophrenie ganz oder zumindest teilweise erfassen. Dabei sind das halbstrukturierte qualitative und halbquantitative psychometrische Interview EASE (Examination of Anomalous Self-Experience) von Parnas et al. [26], das halbstrukturierte Interview EAWE (Examination of Anomalous World-Experience) von Sass et al. [27, 28] und die STEP-Skala (Scale for Space and Time Experience in Psychosis) von Arantes-Goncalves et al. [25] zu erwähnen. Obwohl die beiden erstgenannten Interviews in deutscher Sprache zur Verfügung stehen [29], ist die STEP-Skala lediglich in der englischen Version vorliegend. Um die Erfassung des räumlichen und zeitlichen Erlebens bei Patient*innen mit psychotischen Störungen zu ermöglichen, möchten wir mit vorliegender Arbeit eine deutsche Version zur Verfügung stellen. Darüber hinaus soll die STEP-Skala dazu beitragen, subjektives Erleben von Patient*innen mit psychotischen Störungen und moderne bildgebende Neurowissenschaften miteinander zu verbinden. Nicht zuletzt sollte die vorliegende Arbeit auch das Konzept der „Räumlich-zeitlichen Psychopathologie“ („spatiotemporal psychopathology“) [11, 30] vorstellen.

## Material und Methoden

Die STEP ist ein validiertes Messinstrument zur Erfassung des räumlichen und zeitlichen Erlebens, dessen 25 Einzelphänomene („Items“) insgesamt zwei Dimensionen zugeordnet werden (s. Supplement 1). Die einzelnen Items müssen gesondert im Rahmen einer Exploration erfasst werden. Die Bearbeitungszeit für die Gesamtexploration beträgt in der Regel ca. 30–60 min. Für die einzelnen Schritte der Übersetzung der Skala ins Deutsche s. Supplement 2.

## Ergebnisse

Bei der Entwicklung der Items für die STEP-Skala wurden sowohl Arbeiten und Konzepte früherer Autoren (s. Binswanger [31] und Minkowski [32]) als auch Studien und Konzepte gegenwärtiger Autoren (s. Stanghellini [12, 16], Fuchs [29, 33], Parnas [1, 2, 26], Sass [28, 34–38] und Hirjak [39]) berücksichtigt. Die STEP umfasst insgesamt 14 Items zur Erfassung des räumlichen Erlebens und 11 Items zur Erfassung des zeitlichen Erlebens. Die STEP-Skala basiert insbesondere auf den phänomenologischen Interviews EASE [26] und EAWE [28] sowie der Autismus-Ratingskala [40, 41]. Dennoch wurden mehrere Items der STEP-Skala leicht modifiziert. So basieren die Items #1, #2 und #3 der STEP-Subskala Raum hauptsächlich auf qualitativer Forschung zu Leibphänomenen und Intentionalität bei Schizophrenie [39] und Item #12 wurde durch unsere eigenen Beobachtungen und klinischen Erfahrungen inspiriert. Auch die aufgeführten klinischen Beispiele in der deutschen Version der STEP-Skala wurden von DH, JD und GN aufgrund ihrer klinischen Erfahrung mit psychotischen Patient*innen formuliert.

Die STEP-Subskala Raum (s. Supplement) beinhaltet die folgenden Items: Item #1 (Hyperreflexivität in Bezug auf die körperlichen Bewegungen; [39]), Item #2 (Körperdiskoordination; [39]), Item #3 (Entkörperung; [39]), Item #4 (Mangel an Intentionalität gegenüber der Außenwelt; [26]), Item #5 (Mangel an innerer Intentionalität; [26]), Items #6 und #7 (Zwischenmenschlicher Abstand zu nah und/oder zu weit; [26, 28, 40]), Items #8 und #9 (Abstand zu physischen Objekten und im Raum; [28]), Item #10 (Fragmentierung des Körpers; [26]), Item #11 (Fragmentierung von Raum- und Welt; [28]), Item #12 (Fragmentierung von Raum- und Selbst; [unpublizierte Daten]), Item #13 (Reizüberflutung; [42]) und Item #14 (räumliche Desorientierung; [42]).

Die STEP-Subskala Zeit (s. Supplement) beinhaltet folgende Items: Item #1 (Gegenwart und Zukunft werden von der Vergangenheit bestimmt; [23]), Item #2 (Zukunftsgerichtetheit bricht zusammen, weil Erinnerungen an die Vergangenheit überwältigend sind; [24]), Item #3 (Zeiterleben der Gegenwart; [24]), Item #4 (Zeiterleben der Zukunft; [24]), Item 5 und 6 (die Zeit vergeht langsamer/schneller; [28]), Item #7 (Fragmentierung der Zeit; [42]), Item #8 (Vorahnungen in Bezug auf die Zukunft; [42]), Item 9 (zeitliche Anisotropie; [26]), Item #10 (das Zeiterleben der Gegenwart wird als viel zu lange empfunden; [unpublizierte Daten]) und Item 11 (Synthese der Zeit; [43]).

Jedes Item der beiden Subskalen ist einzeln definiert und zum besseren Verständnis für Untersucher und Patient*innen mit klinischen Beispielen veranschaulicht. Jedes Item kann in fünf Schweregrade 1 (abwesend) bis 5 (stark ausgeprägt) eingeteilt werden. Die Autor*innen der englischen Originalpublikation konnten in einer gemischten Stichprobe von 36 Proband*innen (9 Patient*innen mit Schizophrenie, 10 Patient*innen mit affektiver Symptomatik und 11 gesunde Kontrollproband*innen) eine gute interne Konsistenz (Cronbach’s *α*) sowohl für die STEP-Subskala Raum (*α* = 0,903) und die STEP-Subskala Zeit (*α* = 0,873) als auch den STEP-Gesamtscore (*α* = 0,94) zeigen.

Die Studie von Arantes-Goncalves et al. [25] fand auch eine signifikante Korrelation zwischen den PANSS-Gesamtwerten und der STEP-Subskala Raum (r_s_ = 0,775; *p* < 0,001) und der STEP-Subskala Zeit (r_s_ = 0,845; *p* < 0,001). Nicht zuletzt konnten die Autoren zeigen, dass fast alle Patient*innen mit Schizophrenie im Vergleich zu Patient*innen mit affektiver Symptomatik und gesunden Proband*innen höhere Werte auf der STEP-Skala erreichten.

## Diskussion

Die hier vorgelegte deutsche Version der STEP-Skala nach Arantes-Goncalves et al. [25] stellt das erste Messinstrument im deutschsprachigen Raum zur spezifischen Erfassung des räumlichen und zeitlichen Erlebens bei Patientinnen mit psychotischen Störungen dar. Die STEP-Skala kann auch zur Verlaufsdokumentation dieser Phänomene im klinischen und wissenschaftlichen Setting eingesetzt werden und stellt aus folgenden Gründen eine wichtige Ergänzung der verfügbaren semistrukturierten phänomenologischen Interviews dar: Erstens, hat die englische Originalversion der STEP-Skala eine hohe Validität und Reliabilität gezeigt. Zweitens, fokussiert sich die STEP-Skala spezifisch auf das Raum- und Zeiterleben von Patient*innen mit psychotischen Störungen. Obwohl ein exakter „Cut-off“-Wert zur Differenzierung zwischen Patient*innen mit hoher bzw. niedriger Symptomlast derzeit nicht existiert, kann die STEP-Skala helfen, Patient*innen mit Schizophrenie von Patient*innen mit vorherrschenden affektiven Symptomen/Störungen zu differenzieren. Dies wird auch dadurch bestätigt, dass bestimmte Items in der Skala sehr gut zwischen schizophrenen und affektiven Psychosen differenzieren. Drittens, es konnte ein signifikanter Zusammenhang zwischen den STEP-Werten und dem Vorhandensein sowie dem Schweregrad psychopathologischer Symptome festgestellt werden. Dies zeigt die Relevanz des räumlich-zeitlichen Erlebens für psychopathologische Symptome ganz im Sinne der Räumlich-zeitlichen Psychopathologie (siehe auch [11]).

Die erste Grundlage für diese Skala bildet die Binswanger-These [31], dass die grundlegende Existenz des Menschen räumlich ist und sich in natürlichen, gestimmten, ästhetischen, technischen und historischen Räumen manifestiert. Nach Binswanger kommt es bei der Schizophrenie zu einer Auflockerung und Verschiebung der Betonung der einzelnen räumlichen Formen der Existenz. Neben Binswanger argumentiert auch Klaus Conrad (1905–1961) in seinem 1958 publizierten Hauptwerk *Die beginnende Schizophrenie. Versuch einer Gestaltanalyse des Wahns* [44], dass bei Schizophrenie der von der eigenen Stimmung geprägte Raum „seine Homogenität, Konsistenz und Großartigkeit verliert, was zu wahnhaften Stimmungen oder zu Offenbarungserlebnissen führen kann“. Das Erleben von Patient*innen mit Schizophrenie ist durch eine abnorme Unschärfe und Fragmentierung der räumlichen Grenzen und des intentionalen Bogens zwischen Selbst, Körper und Welt gekennzeichnet. Dies dient als Grundlage für viele der psychopathologischen Symptome wie Wahnvorstellungen, Ich-Störungen, Reizüberflutung und abweichende Beweglichkeit und Orientierung in der Umwelt. Die Hyperreflexivität in Bezug auf die körperlichen Bewegungen (Item #1) lässt sich auch als Trennung von reflektierendem und basalem Selbst auffassen; die Patient*innen versuchen, die entstandenen Brüche in der Gestaltbildung vertrauter körperliche Bewegungen zu überbrücken und verlorene Selbstkongruenz durch krampfhafte Reflexionsprozesse und Selbstkontrolle zu kompensieren [15]. Sie reflektieren zwanghaft über sonst selbstverständliche und implizite Aspekte ihrer Bewegungen. Die Patient*innen müssen über jeden Bewegungsschritt, den sie z. B. beim Zähneputzen oder Treppensteigen ausführen müssen, nachdenken [15]. Bereits die Durchführung tagtäglicher, einfacher Handlungen und Bewegungen im Raum kann somit problematisch werden. Dies führt zur gestörten Körperkoordination im Raum (Item #2), Selbstentfremdung und Reifizierung der leiblichen Vollzüge (Item #3; [45, S. 325]), bis diese als gänzlich ich-fremd und von außen gemacht (Ich-Störungen) erlebt werden [46].

In Bezug auf das Raumerleben bei psychotischen Störungen zeigen die Resultate der Studie von Arantes-Goncalves et al. [25], dass die Einzelitems #2, #4, #7, #12, #13 und #14 der STEP-Subskala Raum am besten zur Charakterisierung von Patient*innen mit vorherrschenden psychotischen Symptomen geeignet waren. Diese Ergebnisse stimmen auch mit einer neueren Studie von Stanghellini et al. [42] überein. Insbesondere Item #7 könnte der Erfahrung der „Zentralität/Invasivität des peripersonalen Raums“ ähneln [42]. Interessanterweise wiesen Stanghellini et al. [42] darauf hin, dass Patient*innen mit Schizophrenie in der sozialen Interaktion einen größeren interpersonellen Raum benötigen als gesunde Personen [42]. Darüber hinaus könnte Item #13 der „Itemisierung und wahrgenommene Bedeutung“ entsprechen, während Item #14 der „Veränderung der Qualität der Umgebung“ ähneln könnte, wie von denselben Autoren vorgeschlagen [42]. Normale räumliche Eigenschaften, wie die relative Größe oder Form von Dingen, können sich verändern. Einige Patient*innen berichten von einem Gefühl, dass der Raum unendlich ist und sich ewig ausdehnt. Dies wird von einem Gefühl der Verdrängung begleitet. Die Umwelt kann aber auch als eine bloße Ansammlung von Einzelteilen, eine Ansammlung von unzusammenhängenden Details wahrgenommen werden. Darüber hinaus scheinen die Items #2, #4 und #12 der STEP-Subskala Raum mit der Störung des Körpererlebens zusammenzuhängen, die als „innerer Raum“ wahrgenommen wird, der zu Raumstörungen neigt [42].

Die zweite Grundlage für diese Skala bildet die Annahme, dass Patient*innen mit psychotischen Störungen oft nicht in der Lage sind, (a) ihren zeitlichen Kontext auf einer Zeitachse zu verorten, (b) verschiedene Teile der Zeitachse, insbesondere die Vergangenheit oder die Zukunft, einzuschätzen bzw. vorherzusagen, (c) eine angemessene „statische“ Zeit inmitten des Flusses wahrzunehmen und/oder (d) ihre eigene Lebenskontinuität innerhalb einer universellen Zeit zu synthetisieren [47]. Binswanger postulierte, dass Schizophrenie durch die Erfahrung einer zeitlichen Fragmentierung charakterisiert werden kann, während affektive Störungen eher eine pathologische Verlangsamung verschiedener Abläufe erleben. Patient*innen mit Angststörungen leiden unter einer Störungen der Zeitvorhersage. Dabei geht es darum, wie ein früherer Zeitpunkt die möglichen Veränderungen und Ereignisse zu einem zukünftigen Zeitpunkt vorhersagen kann, was eng mit der Erfahrung von Gewissheit/Ungewissheit zusammenhängt. Patient*innen mit Angststörungen leiden aufgrund der veränderten Zeitvorhersage typischerweise unter der Erfahrung von massiver Unsicherheit [48, 49].

Minkowski wurde von Bergsons phänomenologischer Philosophie der Dauer inspiriert und stellte fest, dass es eine irreparable Diskrepanz zwischen der Art und Weise, wie der Mensch die Zeit beschreibt, und der Art und Weise, wie er sie lebt (le temps vécu), gibt. Minkowskis Buch *Le temps vécu* [32] wurde 1971 unter dem Titel *Über den zeitlichen Aspekt des Lebens* [50] ins Deutsche übersetzt. Der zweite Teil des Buches wurde dann 1972 unter dem Titel *Über den zeitlichen Aspekt psychopathologischer Phänomene* veröffentlicht. In Bezug auf die STEP-Skala ist erwähnenswert, dass die Items #3, #5, #7, #8 und #9 der STEP-Subskala Zeiterleben die Patient*innen, die zur Gruppe der Schizophrenie gehören, von den anderen beiden Gruppen unterscheiden. Gerade diese Items entsprechen der ursprünglichen Beschreibung des schizophrenen Autismus durch Minkowski. Aus pathophysiologischer Sicht könnten explizite Störungen des zeitlichen Erlebens und autistischer Rückzug schizophrener Patient*innen eine Reaktion auf basale Störung der Zeitsynchronisierung (innere vs. äußere Zeit) sein.

Zusammengenommen wurden von Binswanger, Conrad und Minkowski beschriebene Veränderungen im Raum-Zeit-Erleben als grundlegende Störung der psychischen Erkrankungen aufgefasst. Wenn dem so ist, sollten sich solche basalen Störungen auch im Gehirn manifestieren. Unter der Annahme, dass Raum und Zeit sowohl von der Erfahrung als auch vom Gehirn als eine Art *„gemeinsame Währung“ *(„common currency“; [51, 52]) geteilt werden, gehen wir davon aus, dass eine veränderte Raum-Zeit-Erfahrung in direktem Zusammenhang mit Veränderungen in den Raum-Zeit-Konfigurationen der neuronalen Aktivität des Gehirns steht, z. B. seiner Topographie und Dynamik. So steht z. B. das Erleben von zeitlicher Fragmentierung und Unregelmäßigkeit bei Schizophrenie in Zusammenhang mit mehr oder weniger entsprechenden zeitlichen Ungenauigkeiten und Unregelmäßigkeiten in der neuronalen Aktivität des Gehirns im Millisekundenbereich sowohl der Phase als auch der Amplitude im EEG-Signal. Darüber hinaus steht das Erleben einer abnormalen Langsamkeit bei Depression mit abnormal langen und langsamen, nach innen gerichteten, vergangenheitsorientierten Gedanken im Zusammenhang mit einer verringerten Geschwindigkeit der neuronalen Aktivität (z. B. der globalen Signaltopographie; [53]). Neben Schizophrenie und Depression können zeitliche und räumliche Anomalien auch als Grundstörung von Angststörungen aufgefasst werden. Patient*innen mit Angststörungen sind durch die Erfahrung einer höchst ungewissen, nicht vorhersehbaren Zukunft gekennzeichnet. Aus neurobiologischer Sicht ist bekannt, dass die kortikalen Mittellinienstrukturen des Gehirns als Teil des Default-Mode-Netzwerks (DMN) eine Schlüsselrolle beim Selbsterleben, Erleben mentaler Eigenschaften/Prozesse und bei mentalen Zeitreisen in Vergangenheit und Zukunft spielen. Die kortikalen Mittellinienstrukturen sowie ihre Konnektivität zur Amygdala und zum dorsolateralen präfrontalen Kortex (DLPFC) zeigen bei Angststörungen eine verminderte Synchronisation untereinander. Dies bedeutet eine verringerte zeitliche Vorhersage der Aktivitäten anderer Regionen, was zu einer erhöhten zeitlichen (und räumlichen) Unsicherheit der neuronalen Aktivität von Mittellinienstrukturen und der Konnektivität zwischen Mittellinienstrukturen, Amygdala und DLPFC führt. Zeitliche und räumliche Ungewissheit mit mangelnder Vorhersage der Zukunft manifestiert sich bei Angst also sowohl auf neuronaler als auch auf Erlebnisebene.

**Figure 1:**
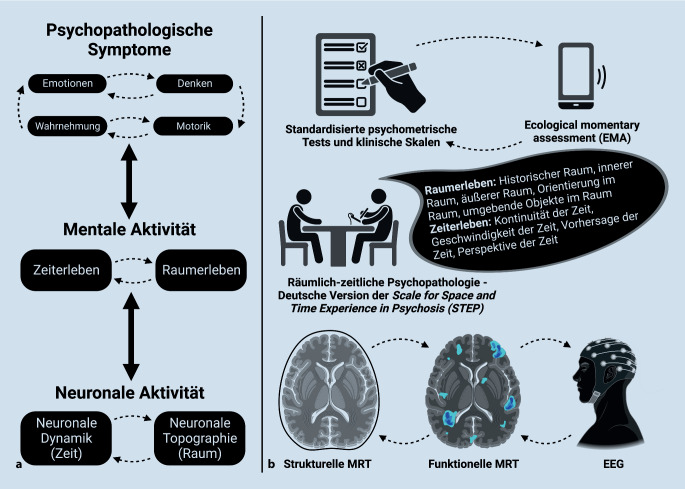

Zusammenfassend lässt sich sagen, dass die räumlichen und zeitlichen Veränderungen sowohl für das subjektive Erleben als auch für das Gehirn als „gemeinsame Währung“ („common currency“) gelten und somit die grundlegende Störung bei Schizophrenie, Depression und Angststörungen darstellen können [11]. Damit verschiebt sich der wissenschaftliche Schwerpunkt auf die Untersuchung zeitlich-räumlicher Strukturen wie Topographie und Dynamik sowohl der mentalen (subjektiven) als auch der neuronalen (objektiven) Aktivität, z. B. von subjektivem Erleben und Gehirnregionen/-netzwerken (Abb. 1). Psychopathologische Symptome können dann als grundlegende Störungen der räumlich-zeitlichen Struktur aufgefasst werden, die sich auf der erlebnisassoziierten und der neuronalen Ebene manifestieren. Ein solcher räumlich-zeitlicher Rahmen öffnet die Tür für krankheitsspezifische Veränderungen im räumlichen und zeitlichen Erleben wie bei Schizophrenie, Depression und Angststörungen. Noch wichtiger ist, dass dieser Ansatz es ermöglicht, das räumliche und zeitliche Erleben als grundlegende Störung mit entsprechenden Veränderungen in den räumlichen und zeitlichen Konfigurationen der neuronalen Aktivität des Gehirns, z. B. seiner Topographie und Dynamik, in Beziehung zu setzen. Zusammengefasst besteht der zentrale Punkt der räumlich-zeitlichen Psychopathologie [51, 52] in einem integrierten Geist-Gehirn-Modell. Gegenwärtig stehen sich die erlebnisbasierte phänomenologische Psychopathologie und biologische Ansätze diametral gegenüber ohne gemeinsame Schnittstelle, da einerseits die Verbindung vom Erleben der Symptome zum Gehirn fehlt und andererseits die neuronalen Veränderungen im Gehirn nicht mit den Strukturen des Erlebens verknüpft werden können. Dies ist der Punkt, wo die Räumlich-zeitliche Psychopathologie eine integrierte Verknüpfung zwischen dem Erleben der Symptome und Gehirnprozessen herstellt: Beide sind über die räumlich-zeitlichen Strukturen miteinander verknüpft. Neuronale Topographie und Dynamik schlagen in mentale Topographie und Dynamik um – die räumlich-zeitlichen Merkmale des Erlebens sind in den räumlich-zeitlichen Prozessen der neuronalen Aktivität des Gehirns manifest und vice versa. Das ist der Kontext in dem die STEP entwickelt wurde, als diagnostisches Instrument für das Raum-Zeit-Erleben in der Schizophrenie im Unterschied zum Raum-Zeit Erleben bei affektiven Störungen und Angsterkrankungen. Dieser Ansatz verspricht in der Zukunft die Entwicklung einer angemessenen Diagnostik und daraus abgeleiteten individualisierten Therapie bei psychischen Erkrankungen.

## Fazit für die Praxis

Störungen des räumlichen und zeitlichen Erlebens wurden bereits Ende des 19. Jahrhunderts im Zusammenhang mit psychotischen Störungen beschrieben und stellen neben Positiv- und Negativsymptomen einen zentralen Aspekt der klinischen Erscheinung dar. Die aktuelle Datenlage ist aber sehr heterogen, was zu einem Großteil auf kleine, inhomogene Stichprobenkollektive und inkonsistente Datenakquisition und -auswertung sowie auf Heterogenität des veränderten räumlichen und zeitlichen Erlebens zurückgeführt werden kann. Vor diesem Hintergrund bedarf es systematischer, prospektiver sowie multimodaler und methodisch ausgereifter Studien, um den Mangel im beschriebenen Forschungsbereich auszugleichen. Künftig könnte die systematische Erfassung des räumlichen und zeitlichen Erlebens in der klinischen Routine wichtige Einblicke in die Pathogenese psychotischer Störungen bieten und erhebliche differenzialdiagnostische, therapeutische und prognostische Konsequenzen zur Folge haben. Hierzu soll die deutsche Version der STEP einen wesentlichen Beitrag leisten.

## Supplementary Information





