# Supplementary material for: Spatiotemporal psychopathology—German version of the Scale for Space and Time Experience in Psychosis (STEP): A validated measurement instrument for the assessment of spatial and temporal experience in psychotic disorders
Source: Nervenarzt. 2023 Jul 10;94(9):835–41. [Article in German] doi: 10.1007/s00115-023-01519-y (PMC10499921; doi:10.1007/s00115-023-01519-y)
Supplement: Supplementary file 1 [file 115_2023_1519_MOESM1_ESM.pdf]

Deutsche Version der Scale for Space and Time Experience in Psychosis (STEP)

Patientennummer:______________

Datum:________________________

Subskala Zeiterleben

Item #1: Gegenwart und Zukunft werden von der Vergangenheit bestimmt

Erläuterung: Gegenwart und Zukunft werden von der Vergangenheit bestimmt, vor allem wegen bewusst erlebter Schuldgefühle bei Depressionen, aus denen ein Bedürfnis nach Bestrafung entstehen kann.

Klinische Beispiele: "Schuldgefühle wegen der Vergangenheit erdrücken mich." | "Ich bin schuldig wegen vieler Dinge in der Vergangenheit." | "Ich muss für vergangene Vergehen bestraft werden." | "Die Zeit ist hoffnungslos." | „Man hängt wie fest und wünscht sich, dass die Zeit schneller vergeht. Die Gedanken sind eng mit Schuld verbunden: Schuld für meine Misserfolge."

Bewertung	Beschreibung
1	Abwesend, wenn definitiv abwesend oder nie erlebt
2	Möglicherweise vorhanden, wenn fraglich vorhanden oder vielleicht erlebt
3	Leicht ausgeprägt, wenn die Vergangenheit das Gefühl hinterlässt, dass die Gegenwart und die Zukunft langsamer erlebt werden, als die inneren Wünsche, die Zeit schneller zu erleben
4	Mäßig ausgeprägt, wenn die Vergangenheit das Gefühl hinterlässt, dass die Gegenwart und die Zukunft langsamer erlebt werden, als die inneren Wünsche, die Zeit schneller zu erleben und es darüber bewusst erlebte Schuldgefühle gibt
5	Stark ausgeprägt, wenn die Vergangenheit das Gefühl hinterlässt, dass die Gegenwart und die Zukunft langsamer erlebt werden, als die inneren Wünsche, die Zeit schneller zu erleben und es ein Bedürfnis nach innerer Bestrafung gibt

Item #2: Die Zukunftsgerichtetheit bricht zusammen, weil die Erinnerungen an die Vergangenheit überwältigend sind

Erläuterung: Es besteht eine Trennung zwischen Vergangenheit und Gegenwart/Zukunft aufgrund eines Übermaßes an traumatischen Erinnerungen in der Vergangenheit oder des Vorhandenseins von Ratlosigkeit und Sinnlosigkeit wegen der Unmöglichkeit das Vergangene zu ändern oder sogar des Gefühls, dass die Erinnerungen an die Vergangenheit nicht mehr präsent sind.

Klinische Beispiele: "Die Vergangenheit hat für mich keine Bedeutung." | "Die Vergangenheit spielt jeden Tag eine Rolle, was für mich sehr belastend ist. Mein Alltag wird von Tausenden von Erinnerungen (an das Erlebte in der Vergangenheit)

beeinflusst". | "Meine Interpretation der Vergangenheit kann nicht geändert werden". |“Es belastet mich sehr, dass ich die vergangenen Entscheidungen und Handlungen nicht rückgängig machen kann.”

Bewertung	Beschreibung
1	Abwesend, wenn definitiv abwesend oder nie erlebt
2	Möglicherweise vorhanden, wenn fraglich vorhanden oder vielleicht erlebt
3	Leicht ausgeprägt, wenn die Erinnerungen an die Vergangenheit als von der Gegenwart und der Zukunft abgekoppelt erlebt werden, weil die Interpretation der Vergangenheit aufgrund eines Übermaßes an traumatischen Erinnerungen nicht geändert werden kann
4	Mäßig ausgeprägt, wenn die Erinnerungen an die Vergangenheit als abgekoppelt von der Gegenwart und der Zukunft erlebt werden, weil die Vergangenheit als sinnlos und verwirrend empfunden wird
5	Stark ausgeprägt, wenn die Erinnerungen an die Vergangenheit als von der Gegenwart und Zukunft komplett getrennt erlebt werden, weil sie als nicht existent empfunden wird

Item #3: Zeiterleben in der Gegenwart

Erläuterung: Die Gegenwart wird als schwierig/problematisch erlebt, wenn es darum geht, ein normales Leben mit anderen zu teilen, als bedeutungslos, mit Schwierigkeiten, nützlich zu sein oder sogar mit beträchtlicher Passivität.

Klinische Beispiele: "Mein großer Wunsch zum aktuellen Zeitpunkt ist es, ein normales Leben zu führen, in dem ich meine Gegenwart mit anderen teilen kann und weniger isoliert bin. " | "Die Gegenwart scheint nutzlos oder unzureichend nützlich und ohne Bedeutung zu sein." | "Ich bin nicht in der Lage, mich auf den gegenwärtigen Moment einzulassen, weil ich ein passiver Zuschauer meines eigenen Lebens bin." | „Ich sitze im Auto als Beifahrer und mein Leben zieht wie ein Film an mir vorbei.“

Bewertung	Beschreibung
1	Abwesend, wenn definitiv abwesend oder nie erlebt
2	Möglicherweise vorhanden, wenn fraglich vorhanden oder vielleicht erlebt
3	Leicht ausgeprägt, wenn der Wunsch besteht, ein normales und gemeinsames Leben mit anderen in der Gegenwart zu führen
4	Mäßig ausgeprägt, wenn die Gegenwart als bedeutungslos und schwer zu verwerten empfunden wird
5	Stark ausgeprägt, wenn der Patient sich als passiver Zuschauer seines eigenen Lebens fühlt

Item #4: Zeiterleben der Zukunft

Erläuterung: Das Denken an die Zukunft wird als sehr schwierig erlebt, vor allem aufgrund von Sorgen und Ängsten, oder weil ihr die persönliche Bedeutung fehlt oder sie sogar als nicht existent empfunden wird.

Klinische Beispiele: "Die Zukunft scheint ohne persönliche Bedeutung zu sein." | "Ich habe keine Perspektive für die Zukunft." | "Wenn ich über meine Zukunft nachdenke, mache ich mir Sorgen und habe Ängste." | „In meiner ersten akuten Phase war ich nur mit den aktuellen Wahninhalten beschäftig. Dabei spielte die Vergangenheit und die Zukunft keine Rolle, es gibt dann nur noch den *aktuellen Drang*.“

Bewertung	Beschreibung
1	Abwesend, wenn definitiv abwesend oder nie erlebt
2	Möglicherweise vorhanden, wenn fraglich vorhanden oder vielleicht erlebt
3	Leicht ausgeprägt, wenn das Denken an die Zukunft Sorgen und Ängste auslöst
4	Mäßig ausgeprägt, wenn die Zukunft ohne persönliche Bedeutung empfunden wird
5	Stark ausgeprägt, wenn die Zukunft als nicht existent empfunden wird

Item #5: Die Zeit vergeht langsamer

Erläuterung: Das Vergehen der Zeit wird als langsamer empfunden, was sich auf verschiedenen Ebenen äußert: langsamer als andere Menschen, langsamer im Allgemeinen und mit dem Gefühl der Ratlosigkeit und Bedeutungslosigkeit, oder sogar komplett angehalten.

Klinische Beispiele: “Die Person erlebt ihre eigenen Handlungen, Gedanken oder Gefühle als unbeweglich oder sehr langsam, während sich Menschen und Ereignisse in einem normalen oder schnelleren Tempo bewegen. " | "Die Zeit fließt für mich nicht. Was hat die Außenwelt mit mir zu tun? Ich stoße nur gegen die Zeit an"; oder: "Ich fühle mich wie ein langsamer, großer Riese." | "Die Zeit wird als eingefroren oder unbeweglich empfunden, ohne dass sie eine Bedeutung hat. Dies kann mit Gefühlen der Ratlosigkeit verbunden sein." | "Die Gedanken standen still, ja alles stand still, als ob die Zeit nicht mehr existierte."

Bewertung	Beschreibung
1	Abwesend, wenn definitiv abwesend oder nie erlebt
2	Möglicherweise vorhanden, wenn fraglich vorhanden oder vielleicht erlebt
3	Leicht ausgeprägt, wenn die Zeit als langsamer empfunden wird als bei anderen Menschen
4	Mäßig ausgeprägt, wenn die Zeit im Allgemeinen als langsamer empfunden wird, mit Ratlosigkeit und Sinnlosigkeit
5	Stark ausgeprägt, wenn die Zeit als stillstehend empfunden wird

Item #6: Die Zeit vergeht schneller

Erläuterung: Die Zeitgeschwindigkeit wird auf verschiedenen Ebenen als schneller empfunden, z. B. schneller als bei anderen Menschen, schneller im Allgemeinen assoziiert mit Ratlosigkeit und ohne Bedeutung oder sogar schneller in jeder Hinsicht.

Klinisches Beispiel: “Die Krankenschwestern und die Patienten bewegten sich schneller als sonst und wenn der Arzt sprach, klang es schnell, laut und in höherer Tonlage.” | „Die Zeit ist schneller vergangen in der Psychose, schnell wie ein Düsenjet.“

Bewertung	Beschreibung
1	Abwesend, wenn definitiv abwesend oder nie erlebt
2	Möglicherweise vorhanden, wenn fraglich vorhanden oder vielleicht erlebt
3	Leicht ausgeprägt, wenn die Zeit als schneller empfunden wird als bei anderen Menschen

4	Mäßig ausgeprägt, wenn die Zeit im Allgemein als schneller empfunden wird, mit Ratlosigkeit und Sinnlosigkeit
5	Stark ausgeprägt, wenn die Zeit immer und egal unter welchen Umständen als schneller empfunden wird

Item #7: Fragmentierung der Zeit

Erläuterung: Die Zeit wird als fragmentiert und in ihrer Synthese als unzusammenhängend erlebt. Dies kann sich darin äußern, dass die Zeit als eine Reihe von Fotografien empfunden wird. Dieses Erleben kann auch mit Gefühlen des Erstaunens, der Ratlosigkeit und der Bedeutungslosigkeit einhergehen.
Klinische Beispiele: "Die Zeit ist wie eine Reihe von Fotos oder Schnappschüssen." | „Ich habe die Zeit wie in einzelnen *Blöcken* erlebt, die teilweise nur wenige Minuten dauern und wie ein *Programm* ablaufen. Aufstehen, Zähne-Putzen und die Zigarette am Morgen gehören zusammen. Danach gibt es einen Bruch. Anschließend Bettmachen, Kaffee und Anziehen. Manchmal gibt es auch von einer Minute auf die andere einen Bruch.“

Bewertung	Beschreibung
1	Abwesend, wenn die Zeit als kontinuierlich erlebt wird
2	Möglicherweise vorhanden, wenn fraglich vorhanden oder vielleicht erlebt
3	Leicht ausgeprägt, wenn die Zeit als eine Reihe von Fotos erlebt wird
4	Mäßig ausgeprägt, wenn die Zeit als Erstaunen und Ratlosigkeit erlebt wird
5	Stark ausgeprägt, wenn die Zeit ohne jede Bedeutung erlebt wird

Item #8: Vorahnungen in Bezug auf die Zukunft

Erläuterung: Die Zukunft wird als eine Reihe von teilweise negativen Vorahnungen erlebt, wobei manchmal etwas Bedrohliches bevorsteht und manchmal die Zukunft als gleichgültig oder sogar nicht existent empfunden wird.

Klinische Beispiele: "Zukünftigen Ereignissen fehlt es an emotionaler Bedeutung oder emotionaler Aufladung." | "...bis zu dem Punkt, an dem es eine Gefahr für seine Existenz darstellt. Er kann Verhaltensweisen beschreiben, mit denen er vermeidet, an die Zukunft zu denken." | "Der Betroffene ist nicht in der Lage, an einen zukünftigen Zeitraum zu denken oder ihn sich vorzustellen oder auch nur die Möglichkeit eines solchen zu begreifen." | „Manchmal hat man den Eindruck zu wissen, was passieren wird. Es ist wie eine Vorahnung von etwas Bedrohlichem und beängstigt mich. Mir ging es schon öfters so, dass ich dann später auch wirklich das erlebt habe, was ich befürchtete.“

Bewertung	Beschreibung
1	Abwesend, wenn definitiv abwesend oder nie erlebt
2	Möglicherweise vorhanden, wenn fraglich vorhanden oder vielleicht erlebt
3	Leicht ausgeprägt, wenn die Zukunft als bedrohlich empfunden wird
4	Mäßig ausgeprägt, wenn die Zukunft als gleichgültig empfunden wird
5	Stark ausgeprägt, wenn die Zukunft als nicht existent empfunden wird

Item #9: Zeitliche Anisotropie

Erläuterung: Anisotropie bedeutet die Richtungsabhängigkeit einer Eigenschaft oder eines Vorgangs. Das bedeutet, dass die Patienten Schwierigkeiten haben, sich gedanklich auf die Vergangenheit, Gegenwart und/oder Zukunft zu fokussieren und diese adäquat zu erleben. Ein weiteres Problem ist, dass die Patienten nicht zwischen Vergangenheit, Gegenwart und/oder Zukunft unterscheiden können, weil die Zeit als fragmentiert oder in ihrem Fluss als verändert erlebt wird. Dies kann sich auch in dem Gefühl äußern, das Leben sei eingeschränkt oder stecke in der Gegenwart fest.

Klinische Beispiele: "Mein Leben ist auf die Gegenwart beschränkt, weil ich mich weder in meine erstarrte Vergangenheit noch in eine Zukunft mit neuen Projekten bewegen kann." | "Es scheint, dass ich immer in der Gegenwart feststecke." | "Es scheint, dass ich immer noch in der Vergangenheit feststecke."

Bewertung	Beschreibung
1	Abwesend, wenn definitiv abwesend oder nie erlebt
2	Möglicherweise vorhanden, wenn fraglich vorhanden oder vielleicht erlebt
3	Leicht ausgeprägt, wenn die Einschränkung mindestens einmal im Monat auftritt
4	Mäßig ausgeprägt, wenn die Einschränkung mindestens einmal pro Woche auftritt
5	Stark ausgeprägt, wenn die Einschränkung mehr als einmal pro Woche auftritt

Item #10: Das Zeiterleben der Gegenwart wird als viel zu lange empfunden ("längeres Jetzt")

Erläuterung: Die Gegenwart ist die dominante Erlebnisform und wird von den Patienten als ausgedehnt und verlängert wahrgenommen. Das aktuelle Erleben steht im Vordergrund, die Vergangenheit und die Zukunft spielen häufig eine untergeordnete oder gar keine Rolle. Dabei kann der Eindruck entstehen, dass mehrere Dinge gleichzeitig passieren, die zeitlich aufeinanderfolgen und normalerweise auch so empfunden werden.

Klinische Beispiele: "Ereignisse in der Zeit, die von anderen Menschen als unterschiedlich wahrgenommen werden, empfinde ich oft als ähnlich und gleichzeitig." | „Kurz vor der hochakuten Phase habe ich den Eindruck, dass mehrere Dinge gleichzeitig passieren. Es gibt da kein Nacheinander, sondern nur noch den *Drang.*" | "Da gibt es nur ein Jetzt, Zukunft und Vergangenheit spielen keine Rolle. Man ist wie in einem Film gefangen."

Bewertung	Beschreibung
1	Abwesend, wenn definitiv abwesend oder nie erlebt
2	Möglicherweise vorhanden, wenn fraglich vorhanden oder vielleicht erlebt
3	Leicht ausgeprägt, wenn diese Erfahrung mindestens einmal im Monat vorkommt
4	Mäßig ausgeprägt, wenn diese Erfahrung mindestens einmal pro Woche vorkommt
5	Stark ausgeprägt, wenn diese Erfahrung mehr als einmal pro Woche vorkommt

Item #11: Synthese der Zeit

Erläuterung: Die Patienten können die Synthese/das Zusammenfügen von Vergangenheit, Gegenwart und Zukunft verlieren. Dies kann sich in dem Gefühl äußern, die gesamte Zeit sei in dem gegenwärtigen Moment konzentriert.

Klinische Beispiele: "Ich habe das Gefühl für die Zeit verloren, weil ich eingeschlafen bin." | "Es scheint, dass die ganze Zeit in dem gegenwärtigen Moment konzentriert ist." | "Ich wusste nicht, wo auf dem Zeitstrahl ich mich befinde." | "Im Nachhinein ist vieles verschwommen, und ich wusste nicht mehr wie lange gewisse Dinge dauerten, ob 1 Tag oder mehrere." | " [Während eines akut psychotischen Erlebnis] hatte ich den Eindruck, dass meine Mutter in einer anderen Zeit war." |"Da gibt es nur ein Jetzt, Zukunft und Vergangenheit spielen keine Rolle. Man ist wie in einem Film gefangen." |„Ich spüre täglich *Risse* in der Zeit, die ich nicht als durchgängig erlebe.“ | „Da werden mir Gedanken aus der Zukunft und der Vergangenheit untergeschoben. Da ist es zum Teil schwierig für mich, festzustellen wer am Hörer ist.“

Bewertung	Beschreibung
1	Abwesend, wenn definitiv abwesend oder nie erlebt
2	Möglicherweise vorhanden, wenn fraglich vorhanden oder vielleicht erlebt
3	Leicht ausgeprägt, wenn diese Erfahrung mindestens einmal im Monat vorkommt
4	Mäßig ausgeprägt, wenn diese Erfahrung mindestens einmal pro Woche vorkommt
5	Stark ausgeprägt, wenn diese Erfahrung mehr als einmal pro Woche vorkommt

Subskala Raumerleben

Item #1: Hyperreflexivität in Bezug auf die körperlichen Bewegungen

Erläuterung: Die Patienten müssen sich intensiv auf jede einzelne Bewegung ihres Körpers konzentrieren, da sie ansonsten nicht fähig wären, ihre Handlungen/Aufgaben zu erfüllen.

Klinische Beispiele: "Ich habe viel über alles nachgedacht, über jede meiner Bewegungen... über Dinge, die man normalerweise als selbstverständlich ansieht, wie zum Beispiel, dass ich jetzt die Zahnbürste nehme..." | „Ich denke beispielsweise, dass ich meinen Fuß oder mein Bein speziell platzieren muss um auf dem Klinikflur entlang gehen zu können.“ | „Ich muss über ganz selbstverständliche Bewegungen richtig viel nachdenken, um sie durchzuführen.“

Bewertung	Beschreibung
1	Abwesend, wenn definitiv abwesend oder nie erlebt
2	Möglicherweise vorhanden, wenn fraglich vorhanden oder vielleicht erlebt
3	Leicht ausgeprägt, wenn dieses Erlebnis mindestens einmal im Monat auftritt
4	Mäßig ausgeprägt, wenn dieses Erlebnis mindestens einmal pro Woche auftritt

5	Stark ausgeprägt, wenn dieses Erlebnis mehr als einmal pro Woche auftritt

Item #2: Körper-Diskoordinierung

Erläuterung: Die Körperbewegungen sind nicht synchronsiert und als ein Ganzes konstitutiert, sondern als Abfolge von einzelnen, fragmentierten Bewegungen mit starren, kantigen und langsamen Zügen/Schritten zusammengesetzt.

Klinisches Beispiel: "Es gab Phasen, in denen ich mich extrem schlecht koordiniert fühlte, als ich nur eine Bewegung mit dem Arm machte und der Arm sich weiter bewegte, als ich es wollte". | „Ich erlebe meine Bewegungen täglich als *sperrig* und *kantig* obwohl ich an und für sich keine Probleme habe die Bewegungen auszuführen.“

Bewertung	Beschreibung
1	Abwesend, wenn definitiv abwesend oder nie erlebt
2	Möglicherweise vorhanden, wenn fraglich vorhanden oder vielleicht erlebt
3	Leicht ausgeprägt, wenn dieses Erlebnis mindestens einmal im Monat auftritt
4	Mäßig ausgeprägt, wenn dieses Erlebnis mindestens einmal pro Woche auftritt
5	Stark ausgeprägt, wenn dieses Erlebnis mehr als einmal pro Woche auftritt

Item #3: Entkörperung

Erläuterung: Manchmal beschreiben sich die Patienten als entleibte Körper *(Cyborgs)* oder körperlose Geister *(Scanner).* Sie erleben sich oft als Roboter oder menschliche Maschinen und werden so zu passiven Zuschauern ihres eigenen Körpers.
Klinische Beispiele: "Der Computer wurde gelöscht und meine Gehirnzellen sind abgestorben. Ich fühle keine Emotionen, weil sie in der rechten Hemisphäre angesiedelt sind und der rechte Computer gelöscht wurde." | "Ich fühle mich schwindlig wie eine Maschine und habe dieses roboterhafte Gefühl in meinem Kopf, als würde ich durch Kameras schauen." | "Ich habe mich wie ein Cyborg gefühlt. Ich war unbeweglich und konnte kaum noch die Treppe hochgehen." | „Die Emotionen waren wie *eingetrichtert* und ich war abgekapselt von der Umwelt.

Bewertung	Beschreibung
1	Abwesend, wenn definitiv abwesend oder nie erlebt
2	Möglicherweise vorhanden, wenn fraglich vorhanden oder vielleicht erlebt
3	Leicht ausgeprägt, wenn dieses Erlebnis mindestens einmal im Monat auftritt
4	Mäßig ausgeprägt, wenn dieses Erlebnis mindestens einmal pro Woche auftritt
5	Stark ausgeprägt, wenn dieses Erlebnis mehr als einmal pro Woche auftritt

Item #4: Mangel an Intentionalität gegenüber der Außenwelt

Erläuterung: Ein durchdringendes oder häufig wiederkehrendes Gefühl von unerklärlicher geistiger oder körperlicher Müdigkeit, Dämpfung der unmittelbaren Lebendigkeit, verminderte Energie, Spontaneität, fehlendem "élan".

Klinische Beispiele: “Ich habe keine Energie, keinen inneren Antrieb." | “Ich fühle mich immer müde und erschöpft." | “Ich habe jede Form der Lust verloren. Ich habe keinen Kontakt zu mir selbst, ich fühle mich wie ein Zombie."

Bewertung	Beschreibung
1	Abwesend, wenn definitiv abwesend oder nie erlebt
2	Möglicherweise vorhanden, wenn fraglich vorhanden oder vielleicht erlebt
3	Leicht ausgeprägt, wenn es jeden Monat auftritt
4	Mäßig ausgeprägt, wenn es jede Woche auftritt
5	Stark ausgeprägt, wenn es nahezu alle Tage oder ständig auftritt

Item #5: Mangel an innerer Intentionalität

Erläuterung: Eine subjektive Störung der Gedankeninitiative, der "Gedankenenergie" oder der intellektuellen Zielsetzung. Schwierigkeiten bei der Planung und Strukturierung einer bestimmten Aufgabe, z. B. beim Kochen oder Schreiben.

Klinische Beispiele: „Ich wollte geistig alles in Ordnung bringen. Ich musste mich sehr anstrengen und konzentrieren um die Aufgabe zu bewältigen.“ | „ein zäheres/visköses Denken, alles kostet Kraft, das Hirn muss schwer arbeiten.“

Bewertung	Beschreibung
1	Abwesend, wenn definitiv abwesend oder nie erlebt
2	Möglicherweise vorhanden, wenn fraglich vorhanden oder vielleicht erlebt
3	Leicht ausgeprägt, wenn es jeden Monat auftritt
4	Mäßig ausgeprägt, wenn es jede Woche auftritt
5	Stark ausgeprägt, wenn es nahezu alle Tage oder ständig auftritt

Item #6: Zwischenmenschlicher Abstand: zu nah

Erläuterung: Das unmittelbare Gefühl, von anderen Menschen vereinnahmt, überrannt, überflutet, eingeengt, oder bedroht zu werden oder sich irgendwie in einer passiven, gefährlich exponierten Position zu befinden und der Gnade anderer Menschen ausgeliefert zu sein. Es kann zu emotionalen Ausbrüchen (Paroxysmen) kommen. Selbst vertraute Menschen können in der Berührung als ungewohnt, fremd und bedrohlich erlebt werden.

Klinische Beispiele: "Die Dinge verschmelzen, es ist als hänge man fest. Andere Personen waren mir zu nah und haben mich bedroht." | „Menschen kommen mir häufig zu nah, meine Körpergrenzen sind offen und verletzlich.“

Bewertung	Beschreibung
1	Abwesend, wenn definitiv abwesend oder nie erlebt
2	Möglicherweise vorhanden, wenn fraglich vorhanden oder vielleicht erlebt

3	Leicht ausgeprägt, wenn nur ein Erlebnis vorhanden ist
4	Mäßig ausgeprägt, wenn zwei Erlebnisse vorhanden sind
5	Stark ausgeprägt, wenn mehr als zwei Erlebnisse vorhanden sind

Item #7: Zwischenmenschlicher Abstand: zu weit

Erläuterung: Die Patienten können sich von anderen Menschen abgetrennt fühlen. Dies kann sich in einem Gefühl der Abgeschiedenheit von anderen Menschen, in einem Gefühl der Ablehnung durch andere oder in dem Gefühl, von anderen ignoriert zu werden, äußern.

Klinisches Beispiel: "Ich habe mich von anderen Menschen abgekapselt und mich in mich selbst verschlossen." | „Ich habe das Gefühl, als wäre ein riesengroßer schwarzer Himmel über mir. Es können Menschen anwesend sein, die ich aber nicht sicher identifizieren kann, da sie weit weg sind. Falls es mir doch gelingt sie zu identifizieren, geht es ihnen schlechter."

Bewertung	Beschreibung
1	Abwesend, wenn definitiv abwesend oder nie erlebt
2	Möglicherweise vorhanden, wenn fraglich vorhanden oder vielleicht erlebt
3	Leicht ausgeprägt, wenn es ein Gefühl der Abgeschiedenheit von anderen Menschen gibt
4	Mäßig ausgeprägt, wenn es ein Gefühl der Ablehnung durch andere gibt
5	Stark ausgeprägt, wenn es ein Gefühl gibt, von anderen ignoriert zu werden

Item #8: Abstand zu physischen Objekten und im Raum: zu weit

Erläuterung: Die Patienten können den Raum und physische Objekte als zu weit entfernt empfinden. Dies kann sich darin äußern, dass sie den Raum und physische Objekte als weiter entfernt, als unendlich oder als unendlich und bedeutungslos empfinden.

Klinische Beispiele: "Unendlicher Raum, unwirklich, wo alles abgeschnitten, nackt und isoliert war." | "Ich fühlte mich verloren und haltlos in der Unendlichkeit des Raumes, die mich trotz meiner Unbedeutsamkeit irgendwie bedrohte." | „Abends beim Einschlafen habe ich die Menschen als Sterne wahrgenommen und konnte telepathisch mit anderen Menschen kommunizieren. Wir schwebten in kleinen Boxen kreisförmig umher, wie Planeten auf ihren Bahnen. Obwohl wir eine Art Schicksalsgemeinschaft darstellten waren wir uns doch auch sehr fern."

Bewertung	Beschreibung
1	Abwesend, wenn definitiv abwesend oder nie erlebt
2	Möglicherweise vorhanden, wenn fraglich vorhanden oder vielleicht erlebt
3	Leicht ausgeprägt, wenn es ein Gefühl des Raums als zu weit entfernt gibt
4	Mäßig ausgeprägt, wenn es ein Gefühl des Raums als unendlich gibt
5	Stark ausgeprägt, wenn es ein Gefühl des Raums als unendlich und bedeutungslos gibt

<u>Item #9: Abstand zu physischen Objekten und im Raum: zu nah</u>

Erläuterung: Der Raum und die Gegenstände werden als zu nah empfunden. Manchmal ist der Patient nicht in der Lage zu bestimmen, wo der Körper endet und die Außenwelt beginnt. Eine körperliche Vermischung, bei der äußere Objekte im Körper des Betroffenen zu sein scheinen oder mit ihm verschmelzen. In manchen Fällen fühlt sich der Patient von der Außenwelt und den Objekten bedrängt.

Klinische Beispiele: "Die Dinge verschmelzen, es ist als hänge man fest. Andere Personen waren mir zu nah und haben mich bedroht." | „Es ist wie ein Hin- und Herschwimmen, der Boden ist wie Wasser und gibt keinen Halt. Die Wände kommen mir immer näher – und zwar exponentiell. Es kommt allerdings nicht zum Knall, sondern fühlt sich so an, als wäre es genau davor und fühlt sich so an als würde ich durch die Wände erdrückt. Wenn ich die Position ändere, lässt das Gefühl nach. Ich weiß nicht genau, wie lange diese Erlebnisse dauern, vermutlich zwischen 10 min. und einer halben Stunde.“ | „Ich *verschmelze* zum Teil mit der Umgebung, meine *Körpergrenzen* sind durchlässig.“

Bewertung	Beschreibung
1	Abwesend, wenn definitiv abwesend oder nie erlebt
2	Möglicherweise vorhanden, wenn fraglich vorhanden oder vielleicht erlebt
3	Leicht ausgeprägt, wenn es ein Gefühl des Raums als zu nah gibt
4	Mäßig ausgeprägt, wenn es ein Gefühl des Raums als verschmolzen mit dem Körper gibt
5	Stark ausgeprägt, wenn es ein Gefühl des Raums als den Körper bedrängend/vereinnahmend gibt

<u>Item #10: Fragmentierung des Körpers</u>

Erläuterung: Dazu kann die Wahrnehmung der Verkleinerung oder Verengung einzelner Körperteile gehören oder die Erfahrung, dass Körperteile oder der ganze Körper dünner, kürzer, größer, zusammengedrückt oder verkleinert werden. Dazu können die Körperteile als fremd, fremdartig, leblos, isoliert, voneinander getrennt, verrenkt oder nicht existent erlebt werden. Dies kann von Gefühlen der Perplexität und Sinnlosigkeit begleitet sein.

Klinische Beispiele: "Es scheint, dass meine Genitalien zu klein sind." | "Wenn ich auf den unteren Teil meines Körpers hinunterschaue, fühlt er sich ständig verdreht und nach links verschoben an, verglichen mit dem Rest des Körpers." | "Ich habe das seltsame Gefühl, dass es der Körper einer anderen Person ist." | „Die *Körpergrenzen der Haut* lösen sich auf. Die Haut ist wechselnd Groß und Klein, pulsierend wie *Strahlenbewegungen* oder *Schwingungen*.“

Bewertung	Beschreibung
1	Abwesend, wenn definitiv abwesend oder nie erlebt
2	Möglicherweise vorhanden, wenn fraglich vorhanden oder vielleicht erlebt
3	Leicht ausgeprägt, wenn Körpterteile als größenverändert erlebt werden
4	Mäßig ausgeprägt, wenn Körperteile als seltsam, fremd, leblos, verrenkt oder nicht existierend erlebt werden
5	Stark ausgeprägt, wenn eine Erfahrung von Fremdheit, begleitet von Ratlosigkeit und Sinnlosigkeit vorhanden ist

Item #11: Fragmentierung von Raum und Welt

Erläuterung: Eine Szene, eine Landschaft oder die sie umgebende Welt verliert ihre kohärente Einheit; Objekte erscheinen isoliert, losgelöst von ihrem Kontext und vielleicht weniger bedeutsam. Die Objekte stehen nicht mehr in Beziehung zueinander oder sind buchstäblich voneinander getrennt. Die Patienten können versuchen, dies zu kompensieren, indem sie versuchen, alles wieder zusammenzufügen.

Klinische Beispiele: "Ich sah die Umgebung nur bruchstückhaft... keine Würdigung des Ganzen... nur Details vor einem bedeutungslosen Hintergrund." | "Ich setzte die Figur in meinem Kopf wieder zusammen. Aber wenn ich mich bewege, entsteht ein neues Bild, das ich wieder zusammensetzen muss."

Bewertung	Beschreibung
1	Abwesend, wenn definitiv abwesend oder nie erlebt
2	Möglicherweise vorhanden, wenn fraglich vorhanden oder vielleicht erlebt
3	Leicht ausgeprägt, wenn die Welt ohne Einheit erlebt wird aber es eine Anstrengung gibt, die Dinge zusammenzusetzen
4	Mäßig ausgeprägt, wenn die Welt ohne Einheit erlebt wird aber es keine kompensatorischen Anstrengung gibt
5	Stark ausgeprägt, wenn die Welt ohne Einheit erlebt wird und es keine kompensatorischen Anstrengung gibt aber begeleitet von Gefühlen der Ratlosigkeit und Bedeutungslosigkeit

Item #12: Fragmentierung von Raum und Selbst

Erläuterung: Emotionen werden in Teile des Körpers wie das Gehirn projiziert und objektiviert. Emotionale Erfahrungen können als Maschine oder Roboter empfunden werden. Aufgrund der Projektion dieser Teile des Selbst kann der Außenraum als vom Patienten abgekoppelt erlebt werden.

Klinische Beispiele: "Meine Emotionen sind vollständig in meiner rechten Hemisphäre angesiedelt." | "Es gibt keinen Unterschied zwischen meinen Gefühlen und einem Roboter oder einer Maschine." | "Ich fühle mich völlig isoliert, wie eine einsame Insel, umgeben vom Meer, ohne jegliche Verbindung zu anderen Ländern."

1	Abwesend, wenn definitiv abwesend oder nie erlebt
2	Möglicherweise vorhanden, wenn fraglich vorhanden oder vielleicht erlebt
3	Leicht ausgeprägt, wenn es die Erfahrung einer Projektion von Emotionen in den Körper oder das Gehirn gibt
4	Mäßig ausgeprägt, wenn die Unterscheidung zwischen den eigenen Emotionen und Maschinen oder Robotern als mangelhaft erlebt wird
5	Stark ausgeprägt, wenn die Trennung von Selbst und Welt aufgrund von Projektionen von isolierten Teilen des Selbst erfahren wird

Item #13: Reizüberflutung

Erläuterung: Die Patienten empfinden den äußeren Raum als zu anregend, zu detailreich und zu überwältigend. Sie fühlen sich verwirrt und desorientiert, weil sie scheinbar eine Reizüberflutung erleben. Die Patienten erleben Reize, die von überall gleichzeitig kommen.

Klinische Beispiele: "Wenn ich den Raum betrete, fühle ich mich überflutet von zu vielen Details, Menschen, Gegenständen und Hinweisen, die von überall her und aus allen Richtungen kommen, die ich nicht alle ordnen und strukturieren kann. Ich werde verwirrt, ängstlich und aufgewühl, weil mir alles unzusammenhängend erscheint." | "Ich kann Reize und Objekte in meiner Wahrnehmung nicht auf Distanz halten, ich kann sie nicht in den größeren räumlichen und zeitlichen Zusammenhang mit anderen Reizen stellen; ich nehme nur noch Details und einzelne Dinge wahr, aber nicht mehr die *Gestalt* oder das Ganze." | "Wenn ich in den Supermarkt gehe, nehme ich nur noch Details von anderen Menschen wahr, aber nicht mehr die *Gestalt* des Supermarkts" | „Ich versuche mich zum Beispiel auditiv abzulenken mit Kopfhörern, sonst würde ich den Weg durch die Stadt nach Hause gar nicht durchhalten und eine ständige *Reizüberflutung* erleben."

1	Abwesend, wenn definitiv abwesend oder nie erlebt
2	Möglicherweise vorhanden, wenn fraglich vorhanden oder vielleicht erlebt
3	Leicht ausgeprägt, wenn ein Übermaß an Reizüberflutung erlebt wird
4	Mäßig ausgeprägt, wenn eine Reizüberflutung erlebt wird und (die Patienten) verwirrt oder ängstlich werden
5	Stark ausgeprägt, wenn eine Reizüberflutung erlebt wird und (die Patienten) aufgewühlt werden

Item #14: Räumliche Desorientierung

Erläuterung: Obwohl sie sich an einem vertrauten Ort oder umschriebenen Standort befinden, fühlen sie sich völlig verloren oder desorientiert. Räumliche Wahrnehmungen werden irreführend. Begleitet werden diese Erfahrungen von subjektiven Zuständen der Desorientierung und Verwirrung.

Klinische Beispiele: "Es gab eine Zeit, in der ich spazieren ging und nicht wusste, wo ich war." | "Ich finde mich nicht zurecht, bin oft orientierungslos und weiß nicht, wohin ich gehen und wie ich mich weiterbewegen soll." | "Ich fühle mich oft wie gelähmt und stecke in bestimmten Positionen und Zeitpunkten fest." | "Ich kann mich nicht entscheiden, wohin ich gehen soll und wie ich gehen soll." | "Ich habe mich damals gefühlt, als wäre ich in einem anderen Raum, im Keller. Dieser Raum war genauso aufgebaut wie das Zimmer in dem ich tatsächlich war, nur, dass er sich in meiner Fantasie unterirdisch befand. Als ich das Zimmer geradeaus verlies, dachte ich, ich wäre eine Treppe hochgestiegen." | "Man geht einen ganz normalen Weg, z. B. den, der nach Hause führt und plötzlich weiß man nicht mehr wo man ist." | „Dieses *Fremdgefühl* ist häufig da, obwohl ich den Ort oder Weg gut kenne. Dies passiert hauptsächlich wenn ich eine Reizüberflutung empfinde. Ich fühle mich dann wie ein *Ping-Pong-Ball.* Ich verspüre eine körperliche Unruhe und Anspannung und habe die Orientierung verloren."

1	Abwesend, wenn definitiv abwesend oder nie erlebt
2	Möglicherweise vorhanden, wenn fraglich vorhanden oder vielleicht erlebt
3	Leicht ausgeprägt, wenn eine Desorientierung und Verwirrtheit erlebt werden
4	Mäßig ausgeprägt, wenn eine Desorientierung, Verwirrtheit und Ambiguität erlebt werden
5	Stark ausgeprägt, wenn eine Desorientierung, Verwirrtheit, Ambiguität und psychomotorische Erregung erlebt werden

Auswertung

Die STEP-Skala mit 25 Items enthält zwei Subskalen, eine für das Zeiterleben (11 Items) und eine für das Raumerleben (14 Items). Die einzelnen Items sind auf einer 5-stufigen Likert-Skala mit 1-5 Punkten zu bewerten, wobei 1 für die Abwesenheit eines Items und 5 für die höchste Ausprägung steht. Somit ergibt sich eine Spannweite von 11-55 Punkten für die Subskala Zeiterleben, eine Spannweite von 14-70 Punkten für die Subskala des Raumerlebens und eine Spannweite von 25-125 Punkten für den Gesamtwert beider Skalen. Je höher die Subskalenwerte und der Gesamtwert ausfallen, desto mehr Auffälligkeiten in Bezug auf das zeitliche und räumliche Erleben liegen vor.
