# Supplementary material for: Spatiotemporal psychopathology—German version of the Scale for Space and Time Experience in Psychosis (STEP): A validated measurement instrument for the assessment of spatial and temporal experience in psychotic disorders
Source: Nervenarzt. 2023 Jul 10;94(9):835–41. [Article in German] doi: 10.1007/s00115-023-01519-y (PMC10499921; doi:10.1007/s00115-023-01519-y)
Supplement: Supplementary file 2 [file 115_2023_1519_MOESM2_ESM.pdf]

# Supplement 2

## Methoden

Die Übersetzung der englischen Version ins Deutsche fand in mehreren, aufeinander aufbauenden Schritten entsprechend den Richtlinien für die Übersetzung fremdsprachlicher Messinstrumente [1] statt (ähnliche Übersetzungsmethodik wurde bereits mehrmals [2-4] angewandt): Die 25-Punkte-STEP-Skala von Arantes-Goncalves et al. [5] (Originalversion ist in der englischsprachigen Publikation [5] zu finden) wurde nach Autorisierung durch F. Arantes-Goncalves und G. Northoff (Letztautor der englischen Originalpublikation) von zwei Fachärzt*innen für Psychiatrie und Psychotherapie (DH und KMK) und einem Arzt mit Psychiatrieerfahrung (JD) ins Deutsche übersetzt. Die Muttersprache der Ärzt*innen ist Deutsch, alle drei haben unabhängig voneinander die STEP übersetzt. Anschließend wurde die deutsche Fassung von zwei bilingualen (Englisch und Deutsch) Personen ins Englische zurückübersetzt und mit dem Original verglichen. Abschließend wurde die jetzt vorliegende modifizierte Fassung des Fragebogens einer Assistenzärztin (MK) im ersten Jahr und zwei Medizinstudentinnen im praktischen Jahr vorgelegt. Die Kolleginnen sollten die Verständlichkeit und alltagssprachliche Angemessenheit der Übersetzung beurteilen und ggf. Veränderungsvorschläge machen [4]. Die Ergebnisse dieser Runde bekräftigten die Übersetzung und deuteten auf eine gute alltagsprachliche Akzeptanz [4].

## Referenzen


1. Schmitt, M. and M. Eid, *Richtlinien für die Übersetzung fremdsprachlicher Messinstrumente.* Diagnostica, 2007. **53**(Heft 1): p. 1-2.
2. Hey, C., et al., *[Penetration-Aspiration Scale according to Rosenbek. Validation of the German version for endoscopic dysphagia diagnostics].* HNO, 2014. **62**(4): p. 276-81.
3. Preuss, U.W., et al., *[Psychometric evaluation of the German version of the Barratt Impulsiveness Scale].* Nervenarzt, 2008. **79**(3): p. 305-19.
4. Kohlman, T., M. Bullinger, and I. Kirchberger-Blumstein, *Die deutsche Version des Nottingham Health Profile (NHP): Übersetzungsmethodik und psychometrische Validierung.* Sozial und Präventivmedizin, 1997. **42**: p. 175-185.
5. Arantes-Goncalves, F., et al., *Scale for Space and Time Experience in Psychosis: Converging Phenomenological and Psychopathological Perspectives.* Psychopathology, 2022. **55**(3-4): p. 132-142.
